# Evolution and Diversity of Biosynthetic Gene Clusters in *Fusarium*

**DOI:** 10.3389/fmicb.2018.01158

**Published:** 2018-06-05

**Authors:** Koen Hoogendoorn, Lena Barra, Cees Waalwijk, Jeroen S. Dickschat, Theo A. J. van der Lee, Marnix H. Medema

**Affiliations:** ^1^Bioinformatics Group, Wageningen University, Wageningen, Netherlands; ^2^Biointeractions and Plant Health, Plant Research International, Wageningen University and Research, Wageningen, Netherlands; ^3^Kekulé-Institut für Organische Chemie und Biochemie, Rheinische Friedrich-Wilhelms-Universität Bonn, Bonn, Germany

**Keywords:** biosynthetic gene cluster, *Fusarium*, koraiol, ancestral state reconstruction (ASR), supernumary chromosome

## Abstract

Plant pathogenic fungi in the *Fusarium* genus cause severe damage to crops, resulting in great financial losses and health hazards. Specialized metabolites synthesized by these fungi are known to play key roles in the infection process, and to provide survival advantages inside and outside the host. However, systematic studies of the evolution of specialized metabolite-coding potential across *Fusarium* have been scarce. Here, we apply a combination of bioinformatic approaches to identify biosynthetic gene clusters (BGCs) across publicly available genomes from *Fusarium*, to group them into annotated families and to study gain/loss events of BGC families throughout the history of the genus. Comparison with MIBiG reference BGCs allowed assignment of 29 gene cluster families (GCFs) to pathways responsible for the production of known compounds, while for 57 GCFs, the molecular products remain unknown. Comparative analysis of BGC repertoires using ancestral state reconstruction raised several new hypotheses on how BGCs contribute to *Fusarium* pathogenicity or host specificity, sometimes surprisingly so: for example, a gene cluster for the biosynthesis of hexadehydro-astechrome was identified in the genome of the biocontrol strain *Fusarium oxysporum* Fo47, while being absent in that of the tomato pathogen *F. oxysporum* f.sp. *lycopersici*. Several BGCs were also identified on supernumerary chromosomes; heterologous expression of genes for three terpene synthases encoded on the *Fusarium poae* supernumerary chromosome and subsequent GC/MS analysis showed that these genes are functional and encode enzymes that each are able to synthesize koraiol; this observed functional redundancy supports the hypothesis that localization of copies of BGCs on supernumerary chromosomes provides freedom for evolutionary innovations to occur, while the original function remains conserved. Altogether, this systematic overview of biosynthetic diversity in *Fusarium* paves the way for targeted natural product discovery based on automated identification of species-specific pathways as well as for connecting species ecology to the taxonomic distributions of BGCs.

## Introduction

The *Fusarium* genus is an extensive fungal genus of ascomycetes consisting of mostly saprotrophic soil-borne species. However, some *Fusarium* species are important plant pathogens. A survey, distributed to fungal pathologists, placed two *Fusarium* species in the top five of fungal plant pathogens based on scientific and economic importance (Dean et al., 2012). In addition, some *Fusarium* species may cause animal and human infections (Nucci and Anaissie, 2007). So while most of the species in the genus are harmless, the ones that actually show pathogenic abilities cause alarming levels of financial as well as clinical damage. Infection of wheat and barley by *Fusarium graminearum*, for example, causes fusarium head blight, FHB (Windels, 2000; McMullen and Stack, 2011). This disease eventually causes kernels to shrivel. When infected kernels are used as seeds for new generations of wheat plants, growth is severely hindered, and final production gravely decreased. Reports have estimated that financial damage from *F. graminearum* exceeds tens of millions of dollars *per annum* (Windels, 2000; Wu, 2007). Apart from the decreased kernel quality, these kernels often contain high amounts of mycotoxins. When consumed, these mycotoxins cause serious health hazards in humans and other animals, ranging from irritations to death.

A range of fungal specialized metabolites have been described in *Fusarium* (Marasas et al., 1979; Emery, 1980; Wiebe and Bjeldanes, 1981; Bacon et al., 1996; Logrieco et al., 1998; Linnemannstöns et al., 2002; Malz et al., 2005; Oide et al., 2006; Troncoso et al., 2010; Yoshida and Nakajima, 2010; Studt et al., 2012; Brock et al., 2013; Cruz et al., 2013; Kakule et al., 2013; Sørensen et al., 2013, 2014; Yin et al., 2013; Jørgensen et al., 2014; Niehaus et al., 2014; Arndt et al., 2015; Von Bargen et al., 2015; Burkhardt et al., 2016; Janevska et al., 2016, 2017; Johns et al., 2017; Reynolds et al., 2017; Wollenberg et al., 2017). Beside functions as mycotoxins, other fungal specialized metabolites play roles in host infection, interspecies competition and defense against predators. To achieve the production of these complex molecules, several enzymes cooperate in a pathway to generate the final product. Genes involved these biosynthetic pathways are often located in close proximity to each other, forming a biosynthetic gene cluster (BGC; see Keller et al., 2005). Along with genes responsible for the biosynthesis of the specialized metabolite, genes with regulatory and transport functions are usually also present in these clusters. Bioinformatic tools such as antiSMASH, CASSIS and SMURF make it possible to rapidly and automatically identify these BGCs in fungal genome sequences (Khaldi et al., 2010; Medema et al., 2011; Wolf et al., 2015). However, obtaining a systematic perspective on BGC diversity across multiple genomes has been more challenging. A few years ago, two key studies have appeared that cataloged the diversity of polyketide synthases (PKSs) and non-ribosomal peptide synthetases (NRPSs) in a number of *Fusarium* genomes (Hansen et al., 2012, 2015). Since then, several methods have been developed to compute all pairwise distances between BGCs in a dataset (Cimermancic et al., 2014; Doroghazi et al., 2014), which can be used to generate sequence similarity networks and group BGCs into gene cluster families (GCFs) based on similarities observed across the entire gene cluster. Moreover, the recently launched Minimum Information about a Biosynthetic Gene cluster (MIBiG) repository provides rich reference data to connect these GCFs to known products based on gene cluster homology, even with clusters from different fungal genera.

Here, we apply these techniques to provide a systematic overview of the taxonomic diversity of BGCs with known products as well as uncharacterized BGCs in eight high-quality genomes across the genus *Fusarium* (Niehaus et al., 2016). Based on this overview, we offer a reconstruction of the evolutionary history of BGC repertoires, and identify clues on ways in which BGCs evolve toward potential new functionalities. Specifically, we show that biosynthetic genes on supernumerary chromosomes are functional, and present a case in which at least three copies are found of a terpene synthase gene that is functionally redundant with the chromosomal copy; this indicates that these supernumerary chromosomes can host genes that are free to evolve toward novel functions without loss of the original function. Finally, we provide strategies to leverage the information on BGC taxonomic distribution for targeted natural product discovery. The network-guided prediction of BGC functions based on a combination of ancestral state reconstruction and transcriptomic analysis exploited in this study will deepen our understanding of the different life styles adopted by *Fusarium* species as well as pave the path toward discovery of bioactive compounds.

## Results and discussion

### Identification of a diverse set of high-quality *Fusarium* genomes

A comprehensive study on biosynthetic diversity critically depends on the completeness of gene content and contiguity within the genome sequences chosen for study, especially because absence of a BGC cannot be confidently established in low-quality genome sequences. For this reason, publicly available genomes were assessed for the covered gene space of the genomes and their corresponding structural annotations using BUSCO (Simão et al., 2015). Eight genomes were included in this study, which had BUSCO scores of at least 97% complete genes (Table S1; Cuomo et al., 2007; Ma et al., 2010; Al-Reedy et al., 2012; Gardiner et al., 2012; Moolhuijzen et al., 2013; Wiemann et al., 2013; Vanheule et al., 2016). Additionally, assembly statistics were evaluated; all genomes used in this study had a minimal N50 contig size of 40 kb and a minimal N50 scaffold size of 1.9 Mb (Table S1). A final indication of the completeness of the assemblies is the presence of certain gene clusters in a corresponding genome annotation; in *Fusarium* species, three gene clusters are considered to be conserved in all species of the genus (Wiemann et al., 2013). These clusters encode fusarubins, gibepyrone A and a yet unknown compound (PKS07). For all species passing the first two criteria, all three of these BGCs were identified on single contigs. The eight strains fall into two distinct clades: *Fusarium graminearum* PH-1, *Fusarium culmorum* CS7071, *Fusarium pseudograminearum* CS3096 and *Fusarium poae* fall into the *Fusarium sambucinum* species complex, while the *Fusarium verticillioides* 7600, *Fusarium fujikuroi* IMI58289, and the *Fusarium oxysporum* Fo47 and *lycopersici* 4287 strains fall into the closely related *F. fujikuroi* and *F. oxysporum* species complexes (O'Donnell et al., 2013). The two *F. oxysporum* strains included in this study were notably different: *F. oxysporum* f. sp*. lycopersici* infects tomato, while *F. oxysporum* Fo47 is a biological control strain that grows in association with tomato without causing symptoms and can protect against infection by other *Fusarium* species (Alabouvette et al., 1993).

### Systematic mapping of *Fusarium* biosynthetic diversity

**Figure 1:**
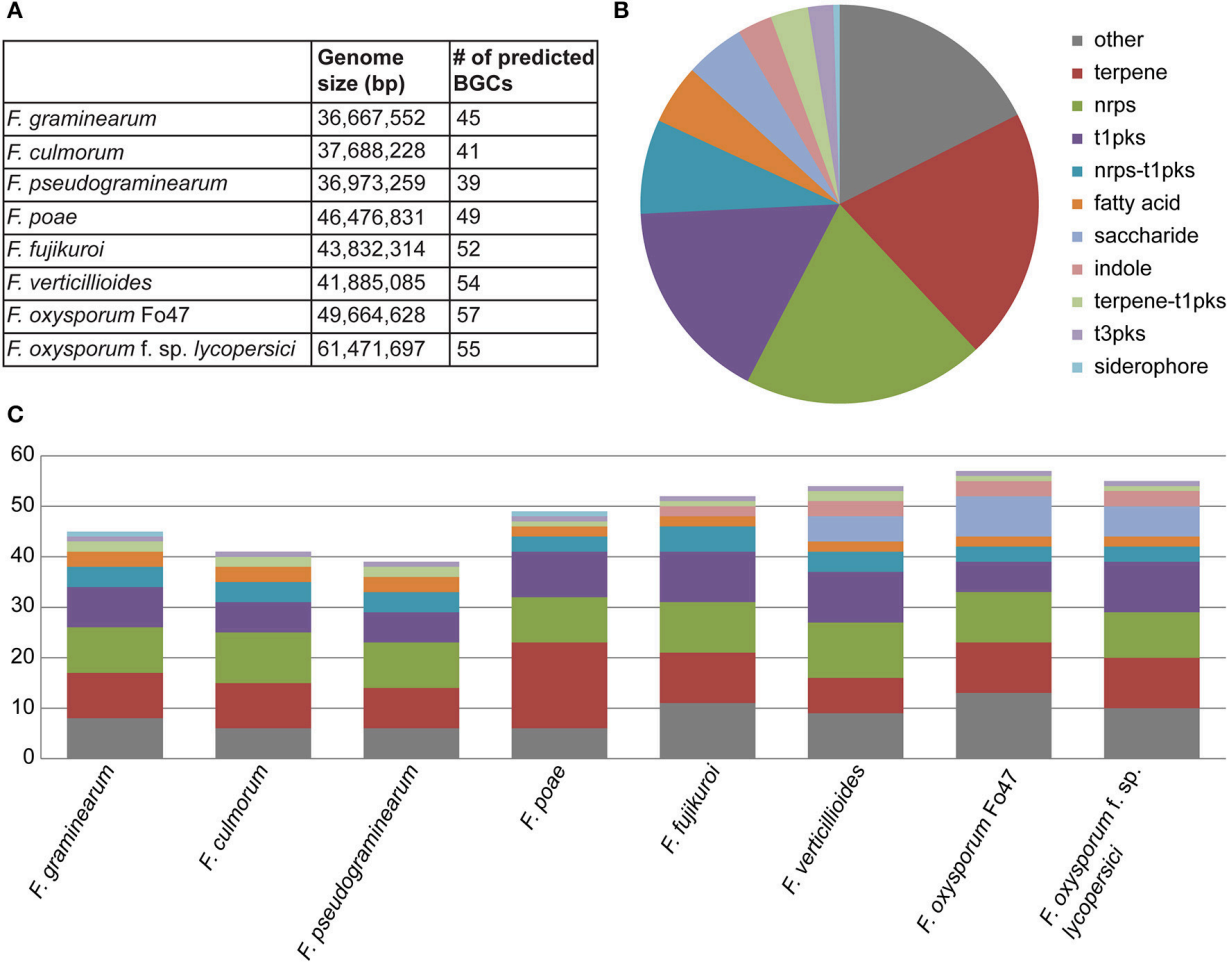
**(A)** Summary of predicted BGCs across all samples (*n* = 392) showing the numbers of biosynthetic gene clusters by organism. **(B)** Pie chart representing the overall distribution of BGC classes across all genomes. **(C)** Composition of BGC classes per genome, showing considerable interspecies variation.

In the eight genomes, a total of 392 biosynthetic gene clusters were found using antiSMASH, averaging 49 clusters per genome, with total numbers ranging from 39 to 57 (Figure 1A). As expected, the most prevalent classes of BGCs were those encoding PKSs, NRPSs and terpene synthases (TSs) (Figures 1B,C). The ClusterFinder algorithm also identified several putative BGCs of unknown function across the eight genomes (Cimermancic et al., 2014). Interestingly, many of these were strain-specific, reinforcing the hypothesis that they carry out specialized metabolic functions specific to certain ecological niches. While most BGCs identified were found on the main chromosomes, several clusters were detected on the supernumerary chromosomes of *Fusarium poae*. Because of the high repeat content of these supernumerary chromosomes, resulting in a fragmentary assembly of these chromosomes, only partial sequences were obtained for these BGCs.

**Figure 2:**
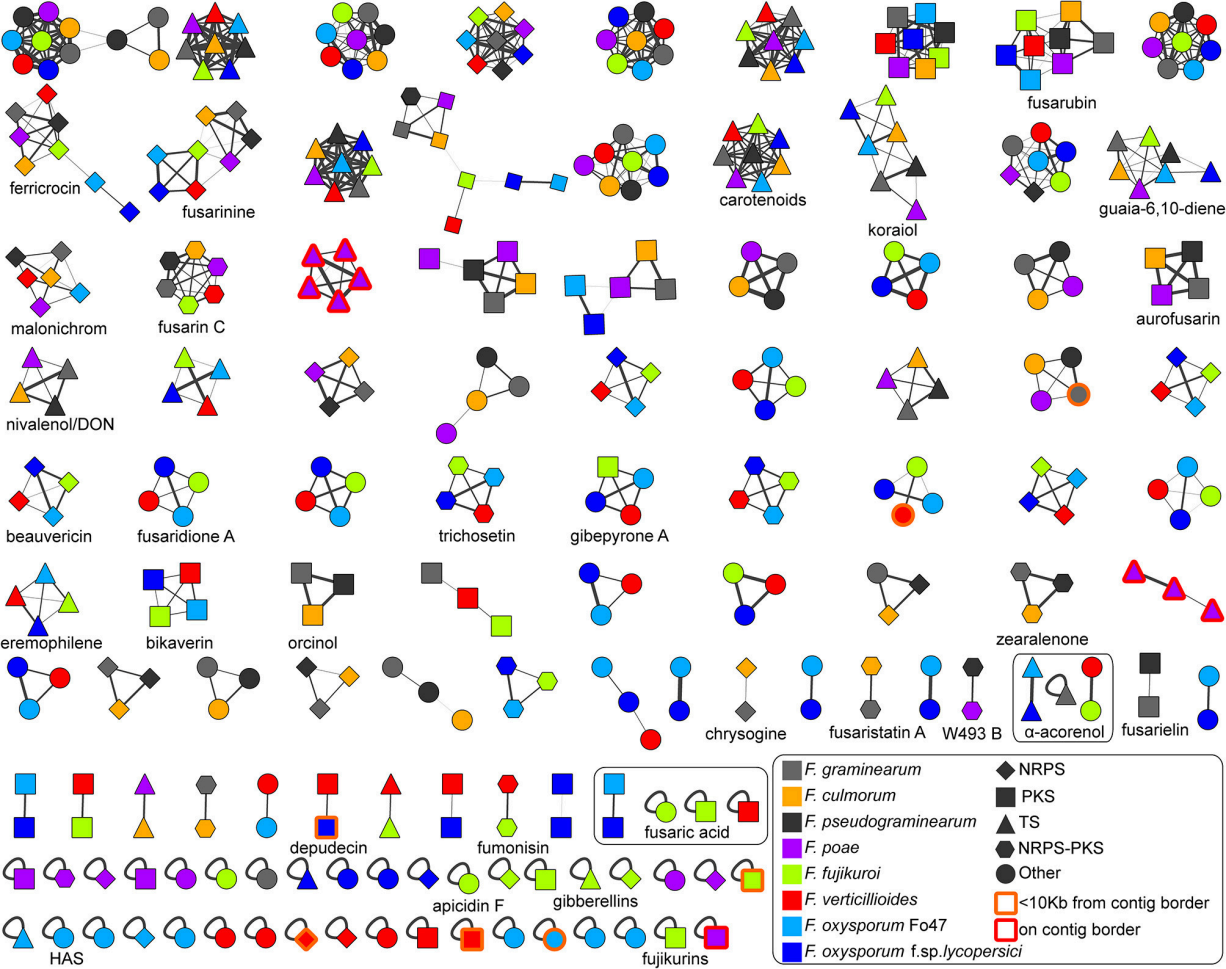
Overview of the *Fusarium* BGC sequence similarity network. The network is displayed at a raw distance cutoff of 0.7. BGCs with known compounds used in following analyses have been marked down in the image. Node colors indicate source genomes, and node shapes indicate biosynthetic classes. The source file including raw data can be downloaded at https://git.wageningenur.nl/hooge096/MSc_Thesis/. The visualization was generated in Cytoscape v3.4.0 (Shannon et al., 2003). The koraiol synthases from *F. poae* studied in detail in the paper are represented by the purple-colored GCFs at the third position of the third row and at the last position of the sixth row.

**Figure 3:**
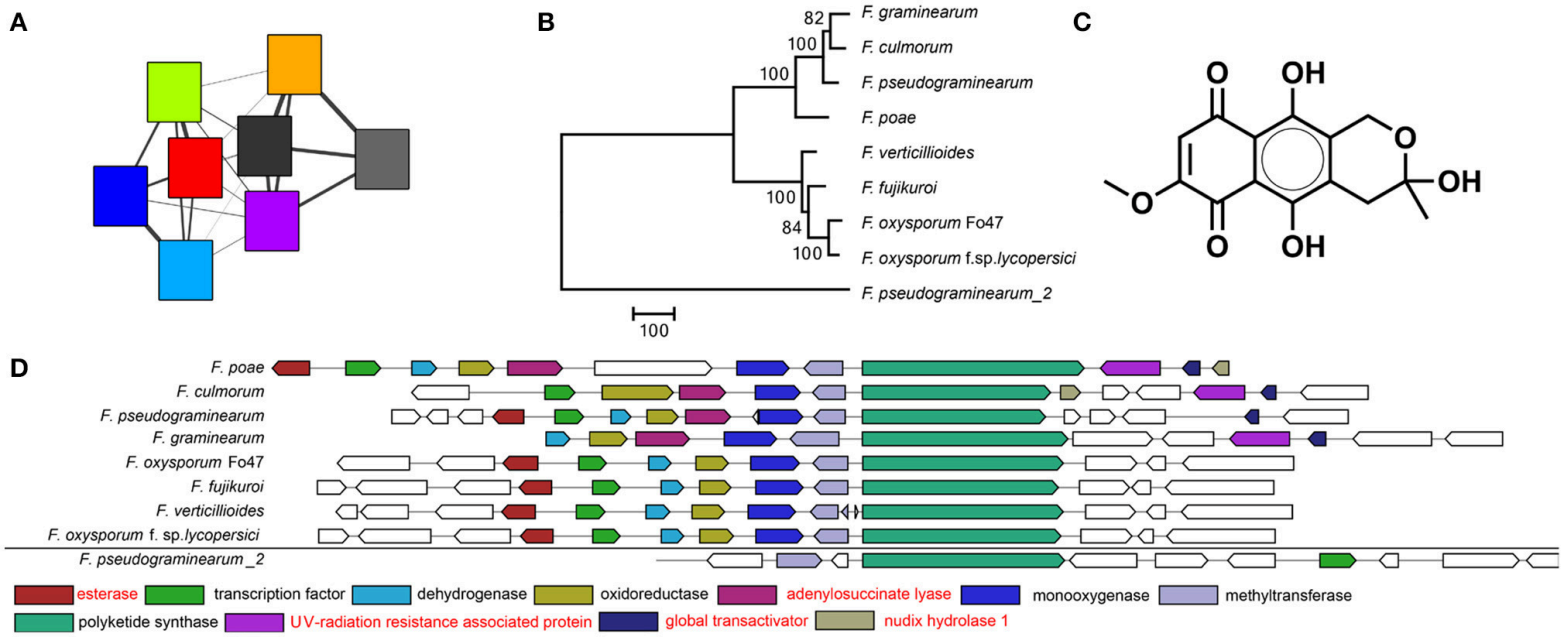
The fusarubin BGC as visualized by **(A)** the sequence similarity network. Colors correspond to the same genomes as in Figure 2. A smaller width of the connecting lines indicates a larger raw distance between two nodes. **(B)** Phylogeny based on the polyketide synthase (PKS) gene shows a large distance between the PKS of *F. pseudograminearum_2* and the other PKSs indicating that its BGC is indeed distantly related and probably not involved in fusarubin production. Bootstrap consensus percentages are shown at nodes in the tree. **(C)** Chemical structure of fusarubin **(D)** Multigeneblast result. The next most similar BGC immediately outside the GCF is indicated at the bottom. Genes with annotations in red are not known to be associated with fusarubin production.

To identify orthology relationships between BGCs across genomes, we reconstructed a BGC sequence similarity network and visualized it using Cytoscape (Figure 2). At a raw distance threshold of 0.7 (see Methods for details on the distance metric), analysis of BGCs with known product shows that the connected components corresponded very well with GCFs for 29 natural products (Marasas et al., 1979; Emery, 1980; Wiebe and Bjeldanes, 1981; Bacon et al., 1996; Logrieco et al., 1998; Linnemannstöns et al., 2002; Malz et al., 2005; Oide et al., 2006; Troncoso et al., 2010; Yoshida and Nakajima, 2010; Studt et al., 2012; Brock et al., 2013; Cruz et al., 2013; Kakule et al., 2013; Sørensen et al., 2013, 2014; Yin et al., 2013; Jørgensen et al., 2014; Niehaus et al., 2014; Arndt et al., 2015; Von Bargen et al., 2015; Burkhardt et al., 2016; Janevska et al., 2016, 2017; Johns et al., 2017; Reynolds et al., 2017; Wollenberg et al., 2017); only the fusaric acid and α-acorenol BGCs were split over a few separate connected components, and comparison with MIBiG reference BGCs (Medema et al., 2015) allowed assignment of 30 GCFs to pathways responsible for the production of known compounds. Per GCF, this assignment was validated by manual analysis based on multiple-BGC alignments generated using MultiGeneBlast (Medema et al., 2013). In complex cases, phylogenetic analysis was performed to validate the GCF assignment (see Figure 3 for an example). Finally, absence of BGCs was re-evaluated by manual BlastP and tBlastN searches with the scaffold biosynthesis enzymes as query, to check for cases in which misannotations, pseudogenization or gene cluster content diversification lead to clusters being missed; this *post-hoc* analysis revealed additional seemingly intact copies of koraiol synthase in *F. verticillioides* and malonichrome synthetase in *F. oxysporum* f. sp. *lycopersici*. It should also be noted that in some cases, BLAST hits were observed with closely related strains, but not with the genome under study; e.g., a malonichrome synthetase homolog was found in *F. fujikuroi* UK99, but not in *F. fujikuroi* IMI58289.

Most of the smaller connected components in the network can be divided into two main groups that correspond to the two major clades in the species phylogeny: the ones that are present in *F. graminearum, F. pseudograminearum, F. culmorum, F. poae* but absent in *F. verticillioides, F. fujikuroi, F. oxysporum* f. sp*. lycopersici, F. oxysporum* Fo47, and vice versa. Increasing the raw distance threshold did not combine these types of clusters, indicating that there is a considerable diversity in BGC repertoires between these two clades.

In all except two cases, GCFs contained maximally one copy per species, indicating that recent whole cluster duplications within genomes were generally absent. One of these exceptions corresponds to terpene BGCs on the supernumerary chromosomes of *F. poae*; a sequence analysis and phylogeny of the terpene synthase sequences in these BGCs showed that these represent paralogous copies of the koraiol sesquiterpene synthase present on the core chromosome (Figure S1**)**. An additional “GCF” was present that contained three small fragments of an additional single koraiol synthase-encoding gene, which had not been fully assembled.

### Identification of functionally redundant sesquiterpene synthases on the supernumerary chromosome(s) of *Fusarium Poae*

The GCFs containing the terpene synthases found on the supernumerary chromosome(s) in *F. poae* were subjected to a more detailed analysis to identify their relation to each other and potentially discover the structure of their products and their functions. All of the identified BGCs on these chromosomes contained the full sequence of a predicted sesquiterpene synthase, sharing 83% identity with the ortholog of the previously identified koraiol synthase from *F. fujikuroi* on the main chromosome of *Fusarium poae* (Brock et al., 2013). Sequence alignment of all predicted terpene synthases revealed that multiple copies of the same terpene synthase are present on the supernumerary chromosomes; however, slight sequence variation between the copies was observed. Sequencing errors or assembly errors were excluded as the cause of these differences by mapping back the original sequence reads to each of the newly identified terpene synthases with a very high stringency (Length overlap ≥ 95%, similarity ≥80%). The frequency with which the differing bases occurred within reads showed that the differences were genuine, and validated the antiSMASH prediction and sequence similarity clustering of the BGCs. From the six valid variants that were discovered, four were synthesized and cloned into an expression vector, which was then transformed into *Escherichia coli* BL21 (Figure S2, Table S2). Three of the four enzymes showed high activity and converted farnesyl diphosphate (FPP) into koraiol as a single product (Figures S3–S6 and Table S3). The incubation with geranyl diphosphate (GPP) yielded a series of monoterpenes for these three enzymes, while geranylgeranyl diphosphate (GGPP) was converted into an unidentified diterpene. In contrast, the fourth enzyme exhibited low activity toward FPP and GPP, and did not accept GGPP. This enzyme has an additional arginine in position 260, located directly in front of the highly conserved NSE triad that is important for functionality of terpene cyclases, which may be the reason for the disturbed enzyme activity. The additional arginine residue could also be responsible for a misfolding of the protein, leading to the observed low expression levels of soluble protein. The presence of at least three functionally redundant copies of koraiol synthase on the supernumerary chromosome reinforces the hypothesis that these genomic elements provide opportunities for evolution to experiment with little cost associated, as the original function remains preserved in the chromosomal copy (Vanheule et al., 2016). The absence of repeat-induced point mutation may also increase the evolutionary rate of protein-coding genes here (Galagan and Selker, 2004). Sometimes this may lead to loss of function (as perhaps in the case of the fourth enzyme above, although evolution toward an unknown function cannot be fully excluded), but it may also lead to neofunctionalization. Further studies on a wider range of *Fusarium* species and BGCs will be required to establish the latter.

### Ancestral state reconstruction sheds light on the evolution of *Fusarium* biosynthetic repertoires

**Figure 4:**
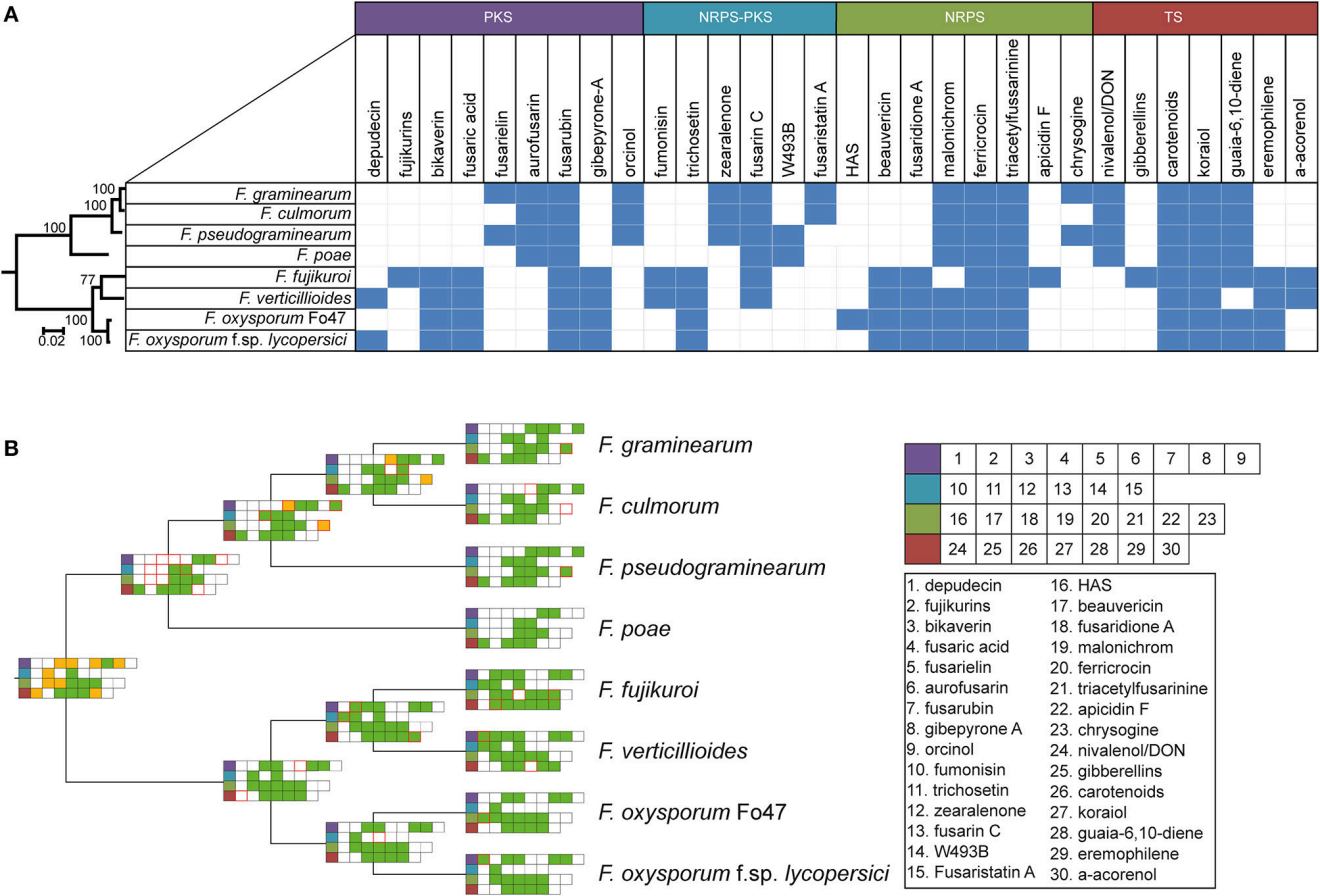
Taxonomic diversity of known BGC repertoires across *Fusarium* **(A)** Binary matrix containing information on the presence or absence of biosynthetic gene clusters with known products in several *Fusarium* species. Color of the products correspond to various specialized metabolite types indicated above these colors. **(B)** Maximum parsimony ancestral state reconstruction of BGCs with an experimentally established product, mapped on the RBP2 cladogram (branch length has no meaning). green = present, white = absent, yellow = unknown, red border = birth/loss event.

Ancestral state reconstruction, the inference of mutations, genome duplications, BGC births and deaths (Proctor et al., 2013) in the past from genomic data obtained from organisms in the present has been an essential technique to increase our understanding of the evolutionary history of a species. Reconstructing the ancestral states of GCFs gives insight into the development of BGC repertoires through time. Focusing on the BGCs for the production of known molecules, we used the GCF assignments to construct a presence/absence matrix of the 30 GCFs to which products were assigned (see details of absence/presence of specific BGCs in Table S4). Subsequently, gains and losses of clusters were inferred using a maximum parsimony approach. To construct a reference phylogeny, the RPB2 gene sequence was used, which corroborated the topology of a previously built, more extensive, phylogeny (O'Donnell et al., 2013). This allowed for reconstruction of the ancestral states of the gene clusters (Figure 4, Figure S7).

The absence/presence matrix and ancestral state reconstruction reveal a number of interesting hallmarks of the evolution of biosynthetic diversity in *Fusarium* species. For example, four GCFs are conserved throughout all genomes, whereas 22 GCFs seem limited to either the genomes of either the *F. sambucinum* species complex lineage or to the *F. fujikuroi* / *F. oxysporum* species complexes; these two groups are also the basal monophyletic groups in species phylogeny. In the case of bikaverin and aurofusarin, both of which are pigments (Malz et al., 2005; Schumacher et al., 2013), this bifurcation is clearly observed (Figure 4A). While their exact roles are not known, these pigments often play a role in protection against environmental conditions (Durán et al., 2002); the two pigments may either be functionally equivalent or provide specific advantages in the varying ecological context where the two groups of fusaria abide. Still other GCFs are limited to just one or two species, and are otherwise only known from different genera, which is suggestive of recent horizontal gene transfer. For example, the BGC for depudecin, which is known to be a virulence factor of *Alternaria brassicicola* in the infection of green cabbage (*Brassica oleracea*) (Wight et al., 2009), follows an interesting pattern: only two species, *F. oxysporum* f. sp. *lycopersici* and *F. verticillioides*, contain this cluster. Interestingly, detailed analysis of the genome of the biocontrol strain *F. oxysporum* Fo47 showed that the cluster appears there in a truncated, most likely non-functional, form. This indicates that the cluster was most likely acquired recently during evolution and partially lost again in some *F. oxysporum* strains, corroborating earlier work by Reynolds et al. (2017). Hence, it may well be (partially) responsible for the difference in lifestyle between these two strains, as *F. oxysporum* f. sp. *lycopersici* is pathogenic to tomato while the biocontrol strain *F. oxysporum* Fo47 is not.

The core enzymes of GCFs seem to follow the *Fusarium* phylogeny derived from RPB2 (see tested examples in Figure S8) or only slightly deviate from it. This indicates that these GCFs were present in the common ancestor of these species and were maintained during evolution. Our study suggests that horizontal gene transfer events occur at a relatively low frequency; still, when they occur, these events may have a large impact. Therefore, correlating these events to species phenotypes may open up new ways for targeted natural product discovery. For example, there are 17 GCFs of unknown function that occur only in one of the two *F. oxysporum* strains; these intriguing candidates may explain adaptations to a specific ecological niche or lifestyle.

### BGC networking allows rapid screening for bioactive compound-synthesizing capacity in *Fusarium* genomes

Fungal BGCs have a wide variety of ecological roles, most of which are not yet fully understood. From the 30 BGCs with known products, only a few are known to be directly involved in pathogenicity. Several mycotoxins are produced, and some of these mycotoxins are involved in pathogenicity toward the host. Depudecin, fumonisin, gibepyrone and fusaric acid have been shown to be virulence factors (Wight et al., 2009; Cruz et al., 2013; Janevska et al., 2016; Studt et al., 2016). Fumonisin, a strong virulence factor for maize, produced by *F. verticillioides*, is not directly related to disease in rice when infected by *F. fujikuroi* (Cruz et al., 2013). This shows that much about its role in virulence is still unknown. By applying sequence similarity network analysis to genome sequences, a quick overview can be acquired concerning the potential for the production of toxins and other important bioactive compounds in a species. For example, *F. oxysporum* Fo47, which is widely used as a biological control strain to prevent other *Fusaria* from infecting a plant, still contains a BGC similar to the one that encodes production of beauvericin in *Aspergillus fumigatus* (Figure S9A). This is a worrisome discovery, since beauvericin production has been found in other *F. oxysporum* strains, and is a mycotoxin capable of triggering apoptosis in several human cell lines (Logrieco et al., 1998). While this compound is not necessarily synthesized by *F. oxysporum* Fo47 under normal conditions, it is possible that through alteration of gene expression through either mutations or changing ecological conditions, production of beauvericin is resumed. We also (for the first time, to our knowledge) detected a hexadehydro-astechrome (HAS) BGC in this *F. oxysporum* strain, showing ±78% sequence identity at the amino acid level with the NRPS identified in *Aspergillus fumigatus* (Figure S9B). Overexpression of this compound is associated with increased mortality in mice when infected by *A. fumigatus* (Yin et al., 2013). While it is thought to only be produced by a small group of opportunistic human pathogenic fungi, the genomic information indicates that a homologous BGC is actually present in *F. oxysporum* Fo47. This should be reason for concern regarding the use of this strain for biological control purposes. Many more of these cases might arise in the future, and preliminary screening of genomes for toxin production capabilities using BGC diversity analyses such as provided here can assist with making decisions concerning widespread use of an organism for either agricultural, biotechnological or medical purposes. Alternatively, they can guide the identification of natural mutants (or the engineering of mutants) that lack such unfavorable traits.

### Network guided re-analysis of RNA-seq data aids identification of specialized metabolites putatively involved in pathogenesis

As shown for the cases of HAS and beauvericin, BGC sequence similarity networks facilitate the identification of BGCs for known molecules in well-studied strains in which these had not been reported before. This also means that previous RNA-seq data can be reevaluated based on this information; moreover, any information from function arising from this may be translated across species. To demonstrate this, an RNA-seq dataset from *F. graminearum* was downloaded from NCBI (Boedi et al., 2016). This dataset was originally used to identify genes involved in pathogenicity, and contained reads extracted from *F. graminearum* growing on living wheat (pth), dead wheat (spr) and a secondary metabolite inducing medium (smi). Additionally, a dataset containing reads from a wild type strain of *F. graminearum* growing on rich medium (wt), as well as a mutant thought to induce trichothecene production (tri) grown on the same rich medium (Zhao et al., 2013) were added to produce the final dataset. Based on analysis of these data (see Methods), we identified genes that were differentially expressed at the statistical threshold of α = 0.01 and were present in the list of anchor genes from the BGCs found in *F. graminearum* (Figure S10). A heatmap of the expression values of the remaining genes was generated and clustered on both axes to reveal patterns of interest (Figure S11**)**. A particular group of interest consisted of the set of anchor genes predominantly active during pathogenic growth: three BGCs from *Fusarium graminearum* producing yet unknown products, along with the BGCs encoding the biosynthetic enzymes for malonichrome and fusaristatin A, as also noted in the previous publication (Boedi et al., 2016). While malonichrome has already been described to be of some importance during infection by regulation of the iron homeostasis in the fungus, the *in vivo* function of fusaristatin A is poorly studied. Interestingly, the BGC for fusaristatin A is also present in *Fusarium culmorum*, prompting further investigation of its possible roles in pathogenicity in this species. The three BGCs producing unknown compounds each have homologs in *F. pseudograminearum* and/or *F. culmorum* according to the network analysis, and have previously been found to be under control by the same transcription factor; *FGP1* (Jonkers et al., 2012; Oide et al., 2015). Mutation studies showed that the *FGP1* gene is essential for virulence, and that Δ*fgp1* mutants are no longer able to infect their hosts. It is likely that the BGCs controlled by *FGP1* play a role in pathogenicity, and therefore are interesting to study in more detail.

## Conclusions

The present work showcases how computational analysis of BGC repertoires using sequence similarity networks and GCF reconstruction allows a systematic analysis of biosynthetic diversity in a genus with a complex specialized metabolome. Moreover, the combination of GCF taxonomic distributions, species phenotypes and ancestral state reconstruction analysis potentially allows for intelligent prioritization strategies to characterize the function of unknown and novel GCFs through synthesis and/or heterologous expression. In this way, the metabolic capacity of a genus can be systematically explored and directly linked to the specific ecologies of its constituent species and strains. This is highlighted by the case of the two *F. oxysporum* strains, one being a pathogen and one a biocontrol strain on tomato, which differ in the presence/absence of depudecin and HAS BGCs, as well as several BGCs of unknown function, which may (partially) explain their large phenotypic differences. The next step will be to extend this kind of analysis to other fungal genera and larger sets of genomes, accelerated through the latest bioinformatic advances. Moreover, the hypotheses generated by these analyses offer promising new avenues for experimental research. Such future studies may lead to new ways to aid in the combat against pathogens, reducing health risks and improving crop yields.

## Materials and methods

### Genome sequences

The genomic dataset consisted of eight *Fusarium* genomes (Table S1). From the GenBank file, FASTA files as well as GFF3 files with gene features were created using custom-made Python scripts to be used as input for BUSCO. To assess genome completeness, BUSCO v3.0.1 was used along with its sordariomyceta_odb9 database. Default parameters were used for all input files, and no additional parameters were used.

### Phylogeny construction

The RBP2 gene was used as the sole gene to base the phylogeny of this *Fusarium* subset on. Using MEGA6, a bootstrapped neighbor-joining tree was constructed using the multiple sequence alignment of the BLAST results from the RBP2 gene in *F. oxysporum* f. sp*. lycopersici* (chromosome 7, location: 3840192-3844693, locus: FOXG_10639). Default settings were used, with 1,000 bootstrap replicates.

### Computational BGC identification

For identification of gene clusters, antiSMASH was used. A development version was used that utilizes ClusterFinder to trim borders of BGCs, in order to enhance sequence similarity networking. Official releases are available at http://antismash.secondarymetabolites.org and development versions are available at https://bitbucket.org/antismash/antismash.

### Sequence similarity network and BGC family reconstruction

AntiSMASH output was used as input to generate a sequence similarity network according to previously published methods (Cimermancic et al., 2014), with a 0.6 weight for the domain duplication index (including sequence identity, according to the same methods as by Cimermancic et al. (2014) but using hmmalign instead of Muscle for multiple-sequence alignment), a 0.2 weight for the Jaccard index and a 0.2 weight for the Goodman-Kruskal gamma index. Singletons were included to ensure that unique BGCs would not be left out in the final network. In order to visualize the networks, Cytoscape v.3.4.0 was used (Shannon et al., 2003). Edge width was set to decrease with an increasing raw distance, to better visualize internodal relations.

### Multigeneblast

To visualize similarity between BGCs in different species, the MultiGeneBlast algorithm was used (Medema et al., 2013). AntiSMASH cluster output files were modified slightly to contain the species name and cluster number in the version/accession feature to make results easier to interpret. The MultiGeneBlast database used for the analysis comprised all predicted clusters across all samples. Predicted clusters were blasted one by one against this database to acquire visual similarity results that could be used to validate GCFs assigned using the sequence similarity network. Default settings were used when running MultiGeneBlast.

### Ancestral state reconstruction

Ancestral state reconstruction of BGCs with known compounds was conducted using Mesquite (Maddison and Maddison, 2016). Since the dataset was small, and maximum likelihood trees can be unreliable and biased in certain cases, a maximum parsimony approach was taken while reconstructing ancestral states (Kolaczkowski and Thornton, 2004). With the use of a phylogenetic tree based on the RBP2 gene, emergence, disappearance and eventual duplication of gene clusters can be traced back through evolutionary history. A binary matrix containing species and gene clusters with known compounds was created to be used in Mesquite.

### Transcriptome analysis

*F. graminearum* RNA-seq reads were mapped back onto the reference genome with the CLC genomics workbench. Preprocessing of reads was also done within CLC genomics workbench, where the last 5% of lowest quality bases were trimmed. If read lengths were shorter than 15 nucleotides after trimming, they were discarded entirely. Reads were mapped using two parameters: %identity and %length overlap. To create the differential expression heatmap, cutoffs used for these were 60 and 80% respectively. Cufflinks was then used to assemble transcripts from the mapped reads to determine expression levels per gene (Trapnell et al., 2010). A final transcriptome was assembled using cuffmerge, which was slightly altered after creation to allow the use of locus tags in following steps instead of the arbitrary IDs that cufflinks assigns to the assembled transcripts. To ensure that replicates of the same sample were consistent with each other, an MDS plot was created (Figure S12). Replicates were expected to end up in close proximity to each other in the graph, while samples were expected to have a larger spacing between them. The plot shows these exact patterns, which leads to the conclusion that the replicates within a sample are consistent with each other, and can be used for a differential expression analysis. Using the original mapped reads with cuffdiff, a differential expression analysis was conducted, which was visualized in R using cummRbund.

### Script availability

All scripts that were created during this project were developed in Python 2.7. Scripts, along with their documentation and data files have been made publicly available on http://git.wageningenur.nl/hooge096/MSc_Thesis.git.

### Gene cloning

All four targeted synthetic genes were amplified from a pUCIDT-AMP plasmid using Q5 polymerase and the primer pairs LB019f_538_48_FPOA_13441 and LB019r_538_48_FPOA_13441 (Table S2). The initial PCR product was elongated by PCR using Q5 polymerase and primer pairs LB020f_538_48_FPOA_13441 and LB020r_538_48_FPOA_13441 to obtain the desired gene carrying homology arms for homologous recombination in *Saccharomyces cerevisiae*. Transformation of *S. cerevisiae* with the elongated PCR products and linearized (XhoI and PvuII digestion) vector pYE-Express was conducted by using the LiOAc/SS carrier DNA protocol. The transformed cells were inoculated on SM-URA agar plates for 3 days at 28°C and plasmid DNA was isolated from grown cells using the kit Zymoprep Yeast Plasmid Miniprep II (Zymo Research). The obtained plasmids were transferred into *E. coli* BL 21 cells by electroporation. The transformed cells were plated on 2YT agar (tryptone 16 g, yeast extract 10 g, NaCl 5 g, agar 20 g, water 1 L, pH 7.2) containing kanamycin (50 mg/L) and grown at 37°C overnight. Single colony cells were picked and inoculated in 2YT liquid medium (10 mL) and grown for 4 h at 37 °C, followed by plasmid isolation using the kit Plasmid Miniprep kit (Promega). The plasmid containing the targeted genes were analyzed by sequencing with t7 promotor primers.

### Gene expression and protein purification

Transformed *E. coli* BL21 mutants were precultured overnight in 2YT liquid medium containing kanamycin (50 mg/L) at 37°C and used for inoculation of 250 mL liquid 2YT medium with kanamycin (50 mg/L). Cells were grown to an OD_600_ = 0.5 at 37°C and 160 rpm. After cooling to 18°C, IPTG (0.4 mm) was added and the cultures were incubated over night at 18°C and 160 rpm. After centrifugation at 4°C and 3,600 rpm for 30 min, the supernatant was discarded and the cell pellet was resuspended in 2 mL binding buffer (20 mm Na_2_HPO_4_, 0.5 m NaCl, 20 mm imidazole, 1 mm MgCl_2_, pH 7.4) and cells were disrupted by ultra-sonication on ice for 5 × 30 s. The cell debris was removed by centrifugation (2 × 10 min) at 4°C and 11,000 rpm to yield the soluble enzyme fractions. The protein was purified by Ni^2+^-NTA affinity chromatography with Ni^2+^-NTA superflow (Qiagen) with binding buffer (4 × 1 mL; 20 mm Na_2_HPO_4_, 0.5 m NaCl, 20 mm imidazole, 1 mm MgCl_2_, pH 7.4) and elution buffer (3 × 1 mL; 20 mm Na_2_HPO_4_, 0.5 m NaCl, 500 mm imidazole, 1 mm MgCl_2_, pH 7.4). The obtained fractions were collected and analyzed by SDS-PAGE (Figure S2). Fractions containing protein of the correct size (elution fraction 1 and 2) were pooled and used for enzyme reactions.

### Enzyme reactions and product analysis

The obtained pooled elution fractions containing proteins of the expected size were aliquoted and mixed with freshly prepared solutions of GPP, FPP and GGPP (0.2 mL, ca. 1 mg/mL in water) and incubated at 28°C for 14 h. The reaction mixture was extracted with hexane (0.2 mL) and the organic layer was separated, dried with MgSO_4_ and directly analyzed by GC/MS. The total ion chromatograms are shown in Figures S3–S5.

### GC/MS analysis

The crude extracts were analyzed by use of an Agilent HP7890B gas chromatograph, fitted with a HP-5MS silica capillary column (30 m, 0.25 mm i. d., 0.50 μm film), connected to a HP5977A mass detector. The GC-MS conditions were as follows: (1) inlet pressure: 77.1 kPa, He flow 23.3 mL min^−1^; (2) injection volume: 1 μL; (3) injection mode: splitless, valve time 60 s; (4) oven temperature ramp: 5 min at 50°C increasing at 5°C min^−1^ to 320°C; (5) carrier gas He at 1 mL min^−1^; (6) transfer line: 250°C; (7) electron energy: 70 eV. Retention indices (*I*) were determined from a homologous series of *n*-alkanes (C_8_-C_40_).

## Author contributions

MHM and TL conceived the project. KH, LB, CW, JSD, TL, and MHM participated in designing the experiments and analyses. KH carried out bioinformatic analysis, under supervision of MHM and TL. LB performed cloning and chemical analysis, under supervision of JSD. KH wrote the first draft of the paper with significant input from LB and JSD. All authors contributed to data interpretation and editing of the paper.

### Conflict of interest statement

MHM is a member of the Scientific Advisory Board of Hexagon Bio. The authors declare that the research was conducted in the absence of any commercial or financial relationships that could be construed as a potential conflict of interest.
