# Supplementary material for: Evolution and Diversity of Biosynthetic Gene Clusters in Fusarium
Source: Front Microbiol. 2018 Jun 5;9:1158. doi: 10.3389/fmicb.2018.01158 (PMC5996196; doi:10.3389/fmicb.2018.01158)
Supplement: Supplementary file 1 [file Data_Sheet_1.docx]

## Supplementary Information

Figure S1. Evolutionary relationships between CDS sequences of koraiol synthases with TPS X being the unknown terpene synthase found on the supernumerary chromosomes of *F. poae*. The phylogeny was constructed using the UPGMA method, and the final tree is based on 1000 bootstrap replications. A different terpene synthase (encoding *a*-acorenol) from *F. fujikuroi* was chosen as outgroup in order to root the tree.

**
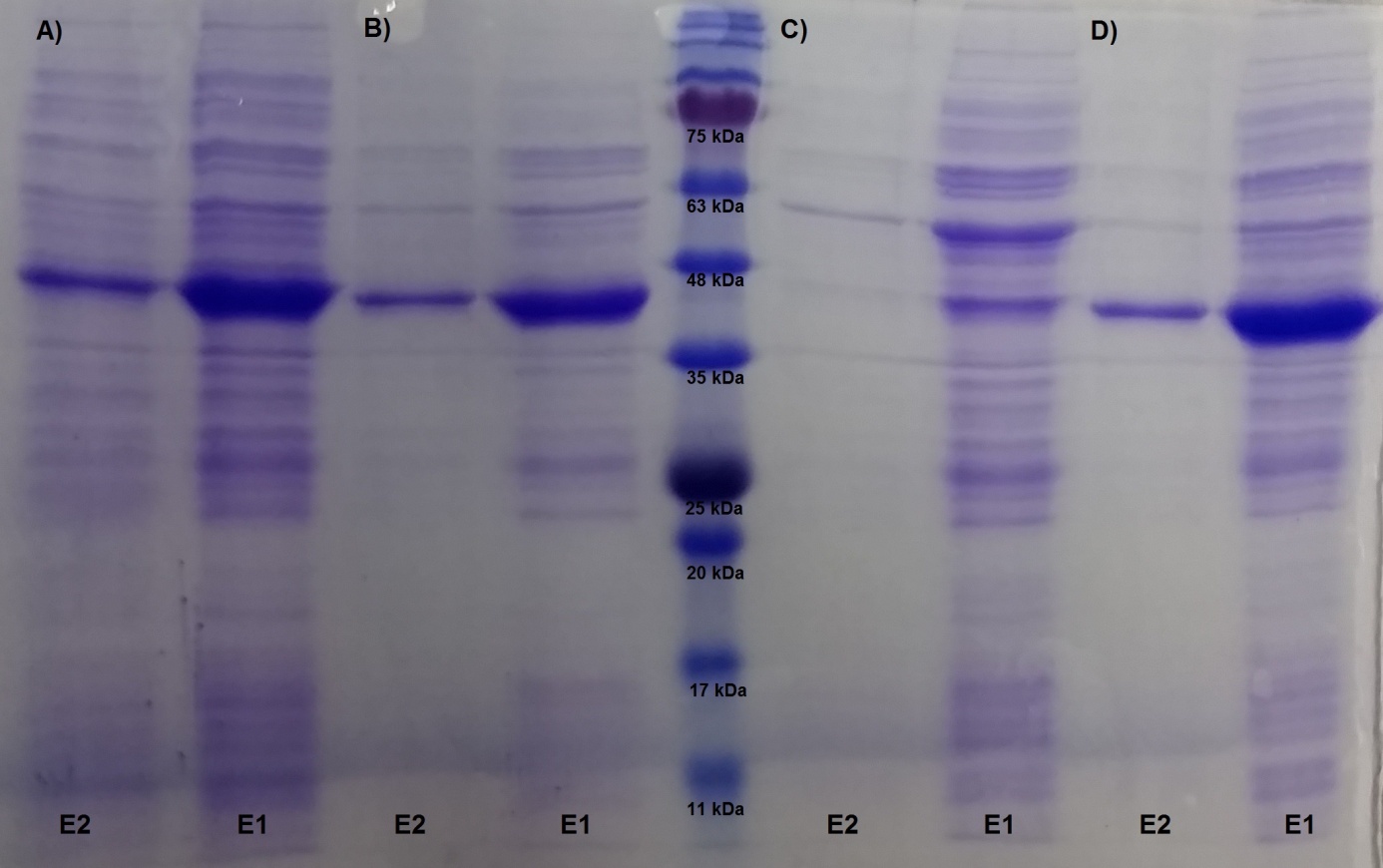
**


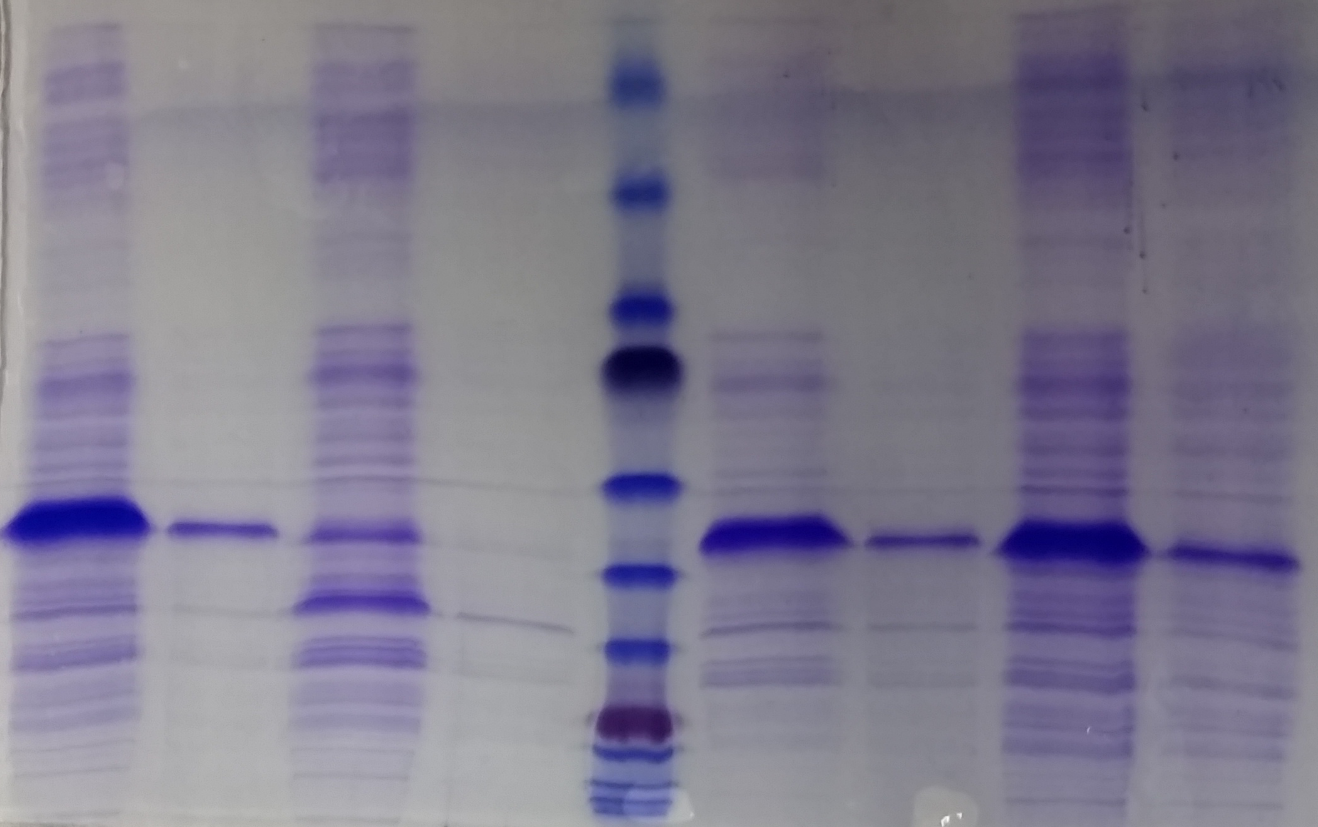


Figure S2. SDS-PAGE analysis of elution fractions E1 and E2 of recombinant proteins. A) TPS X (variant 3), B) 551_43_FPOA_13095, C) 573_45_FPOA_13378, D) 538_48_FPOA_13441. Original image pasted below.


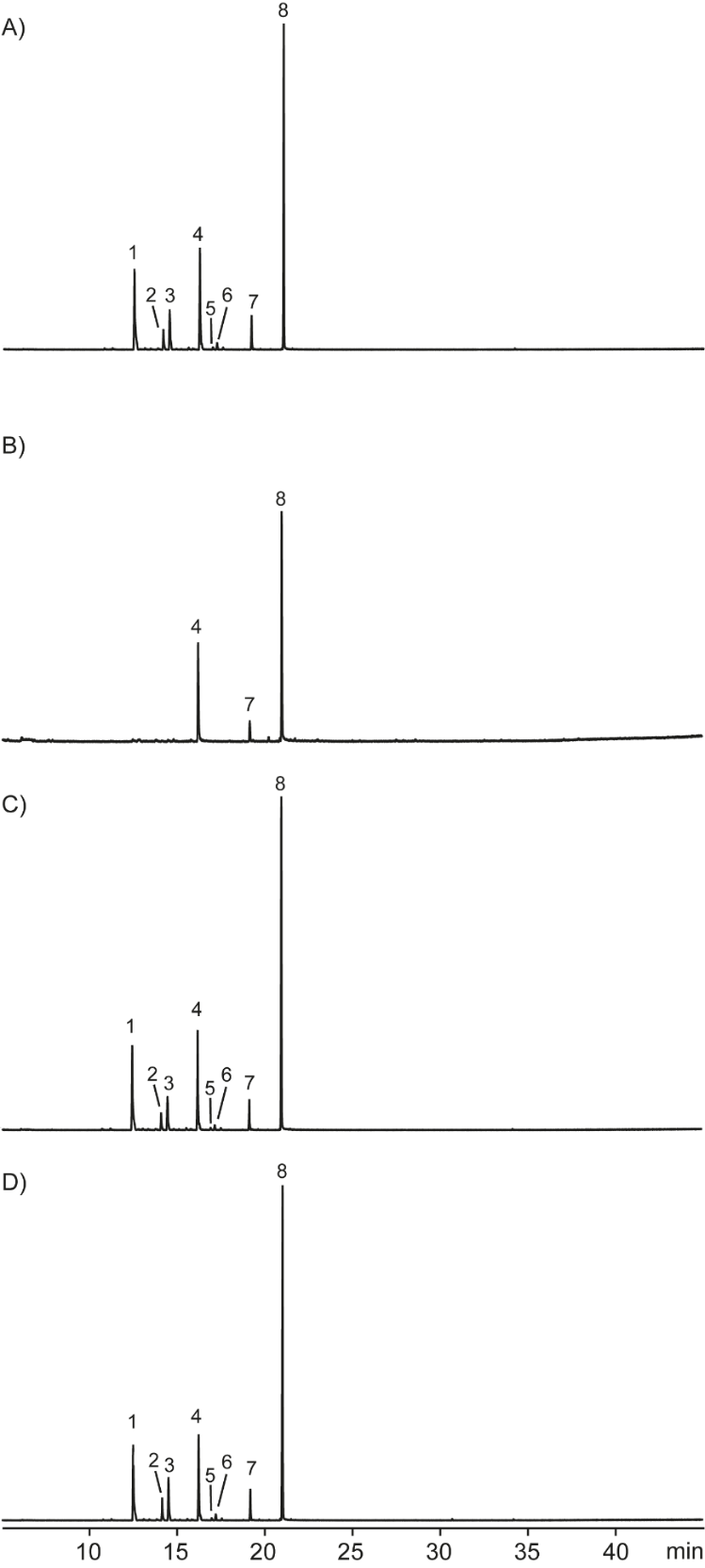


Figure S3. Results of incubation experiments with GPP. Total ion chromatogram for incubation with A) 538_48_FPOA_13441, B) 573_45_FPOA_13378, C) 551_43_FPOA_13095, D) T3_Variant. Peak numbers refer to compound numbers in Figure S6 and Table S3.


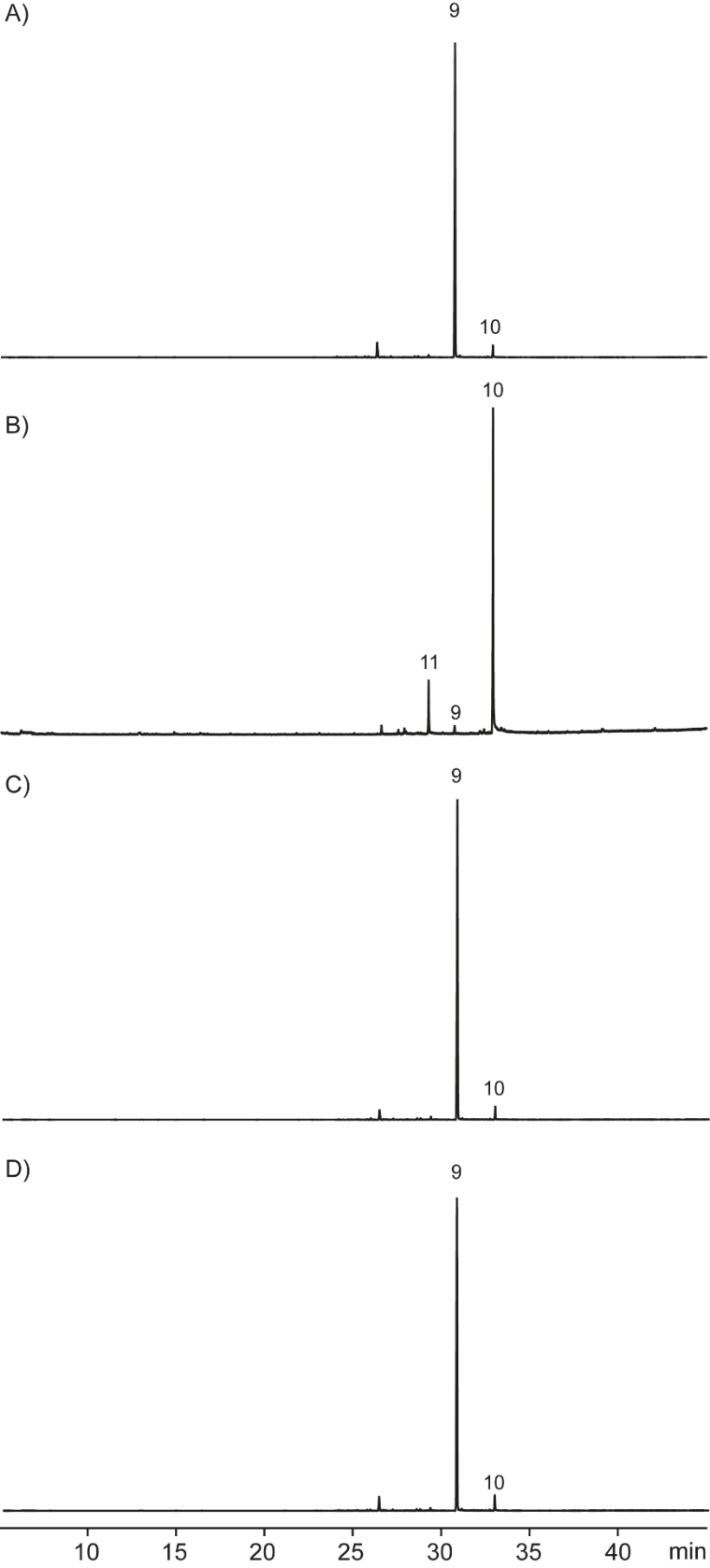


Figure S4. Results of incubation experiments with FPP. Total ion chromatogram for incubation with A) 538_48_FPOA_13441, B) 573_45_FPOA_13378, C) 551_43_FPOA_13095, D) T3_Variant. Peak numbers refer to compound numbers in Figure S6 and Table S3.


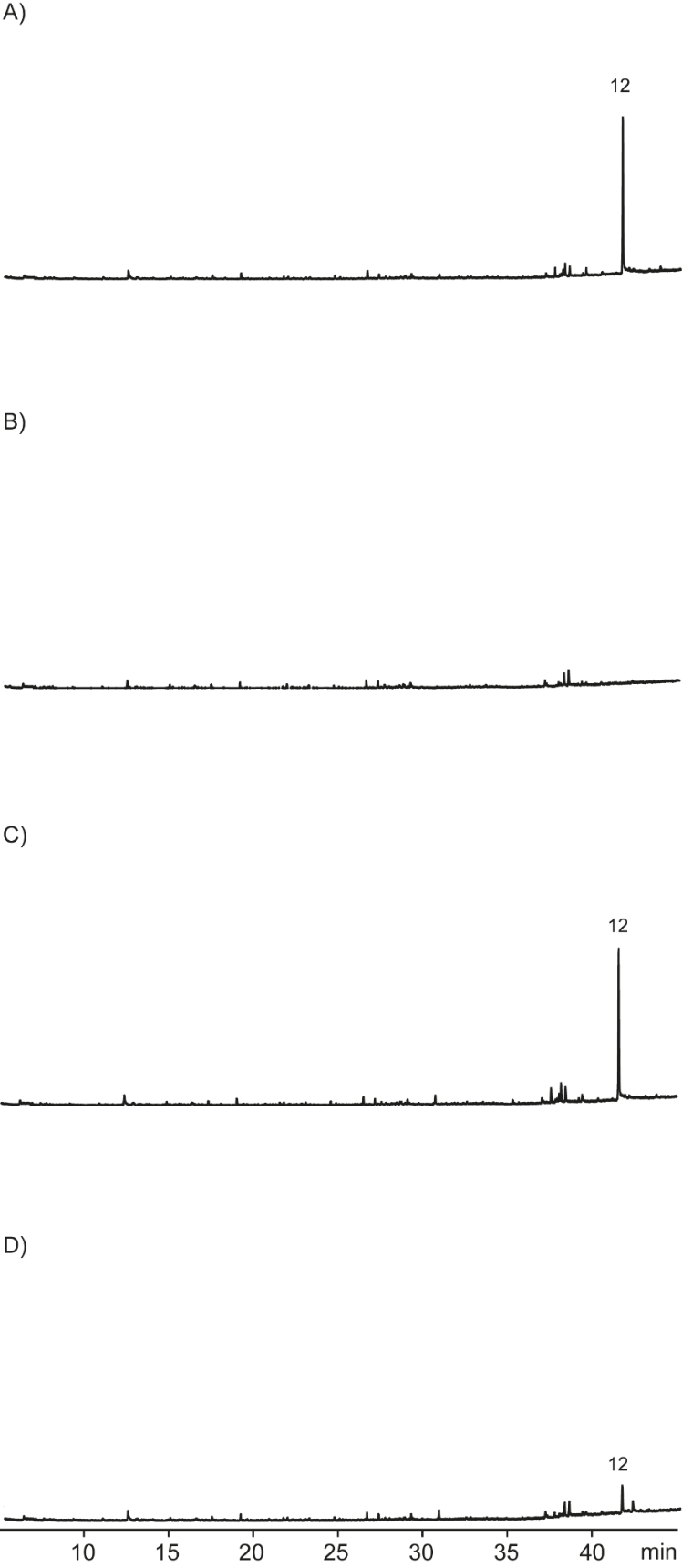


Figure S5 Results of incubation experiments with GGPP. Total ion chromatogram for incubation with A) 538_48_FPOA_13441, B) 573_45_FPOA_13378, C) 551_43_FPOA_13095, D) T3_Variant. Peak numbers refer to compound numbers in Figure S6 and Table S3.

Figure S6 Structures of identified terpenes from enzyme reactions. Compounds 1,2,4,8,10 and 11 are hydrolysis or elimination products of GPP or FPP, respectively.


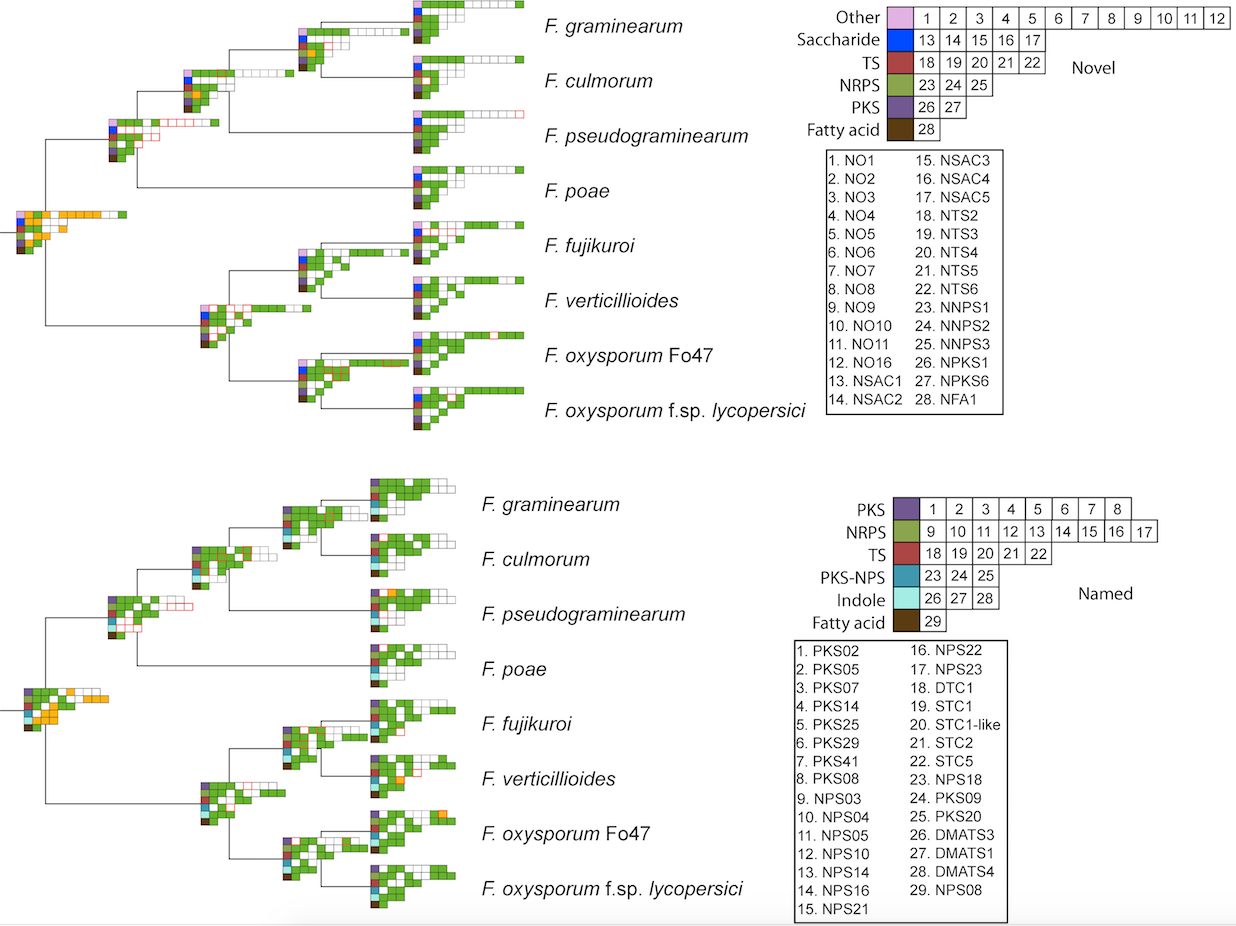


**Figure S7 Taxonomic diversity of BGC repertoires across *Fusarium* without annotated products.** Maximum parsimony ancestral state reconstruction of BGCs mapped on the RBP2 tree where green = present, white = absent, yellow = unknown or fragmented, red border = change. A) Shows the novel BGCs that are not yet described in literature, while B) shows BGCs that were previously described in literature.


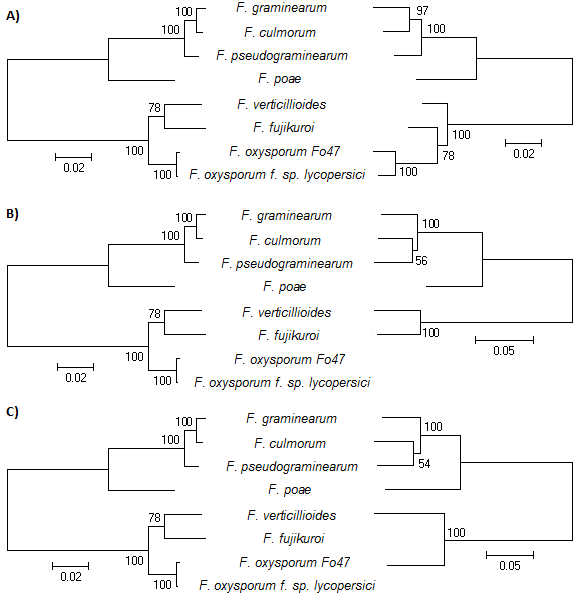


Figure S8 Comparison of the *Fusarium* phylogeny as derived from the RBP2 gene (shown on the left) with those of A) fusarubin, B) fusarin C and C) malonichrome BGC anchor genes (shown on the right). BGC phylogenies are consistent with the *Fusarium* phylogeny

**A)**


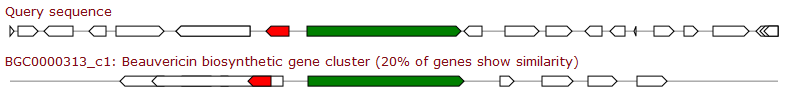


**B)**


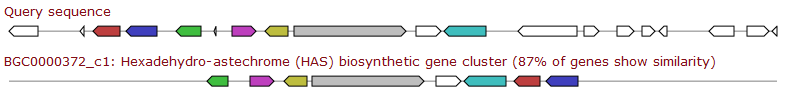


Figure S9 MultiGeneBlast output of A) the beauvericin cluster in *F. oxysporum* Fo47 with its reference in *A. fumigatus* in MiBIG, and B) the HAS cluster in *F. oxysporum* Fo47 with its reference in *A. fumigatus* in MiBIG.


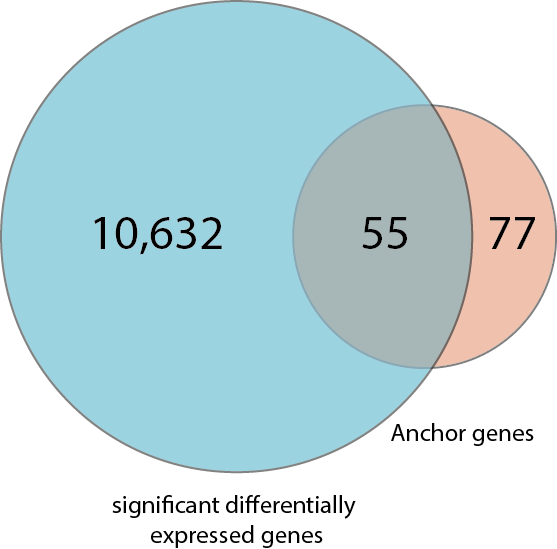


Figure S10 Visualisation of the differentially expressed genes and the genes of interest from the RNA-seq data.


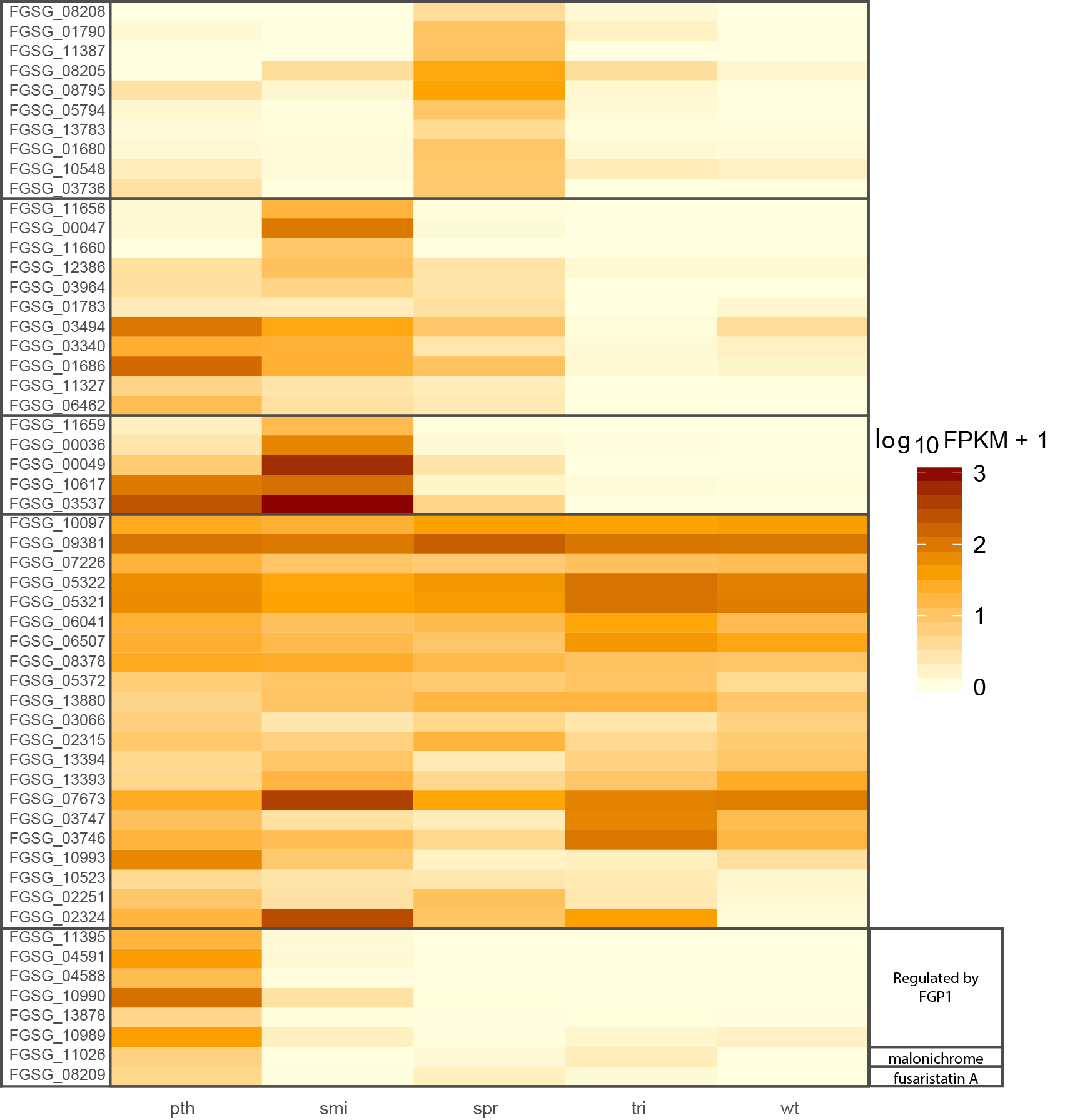


Figure S11 Heat map of expression levels of differentially expressed BGC anchor genes. Clustering based on Jensen-Shannon distance was applied to both axes. Arbitrary categories are indicated with horizontal black lines, with the bottom category being the one that was of most interest. The anchor genes that are regulated by FGP1 belong to BGCs NPS14 (FGSG_11395), PKS29 (FGSG_04591, FGSG_04588) and NPS5/NPS9 (FGSG_10990, FGSG_13787 & FGSG_10989), none of which have an associated product.


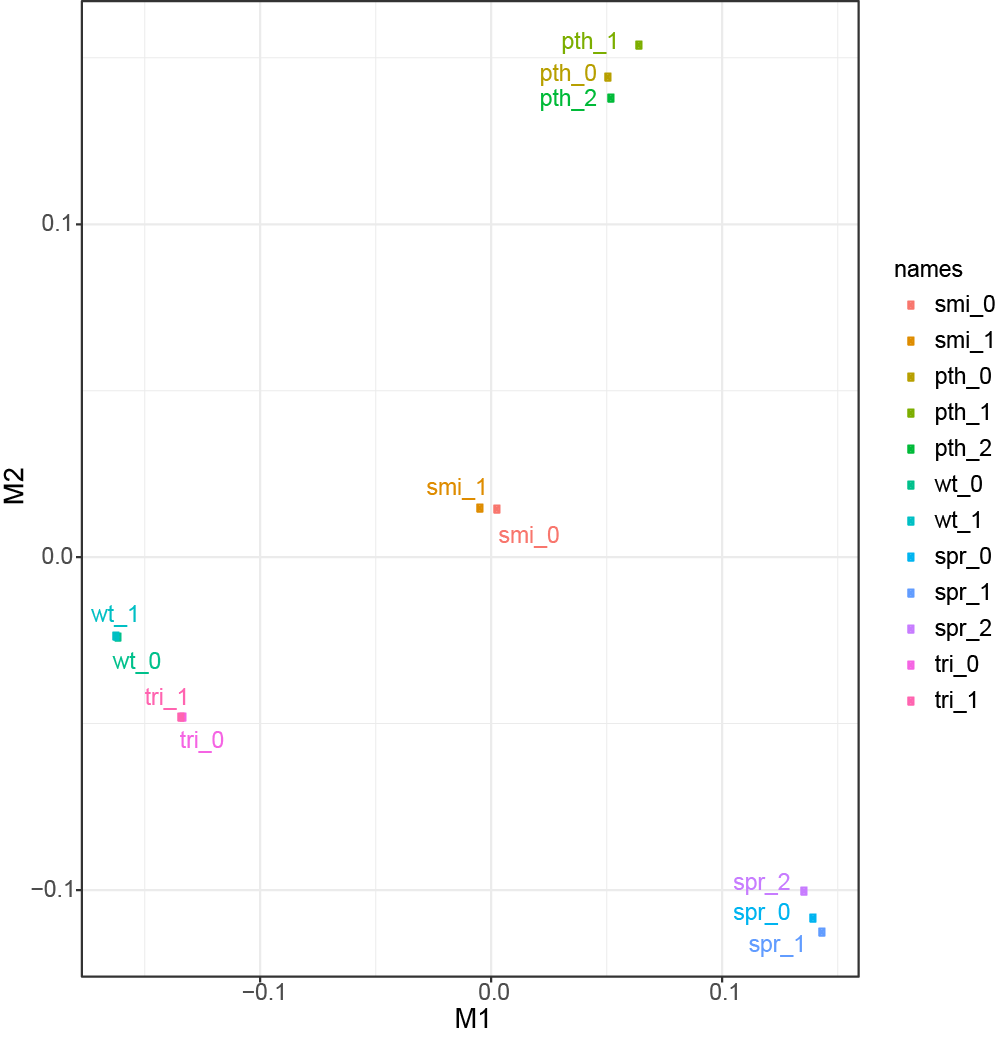


Figure S12 MDS plot of the various replicates showing that the replicates of a single sample cluster together, while individual samples are spaced further apart.

Table S1. Summary of assembly statistics containing N50 and BUSCO scores along with general genome sta­tistics.

| Species | Genome  size (bp) | Scaffolds | Scaffold  N50 (bp) | Contigs | Contig  N50 | BUSCO  Score | NCBI BioProject  Accession |
| --- | --- | --- | --- | --- | --- | --- | --- |
| *Fusarium graminearum* PH-1 | 36,667,552 | 199 | 8,791,613 | 424 | 258,133 | 3644/49/32 | PRJNA13839 |
| *Fusarium culmorum* CS7071 | 37,688,228 | 207 | 8,831,140 | 2,274 | 39,999 | 3685/18/22 | PRJEB1738 |
| *Fusarium pseudograminearum* CS3096 | 36,973,259 | 281 | 8,840,934 | 685 | 186,303 | 3691/14/20 | PRJNA66583 |
| *Fusarium poae* | 46,476,831 | 181 | 8,783,590 | 182 | 9,631,096 | 3691/16/18 | PRJNA319914 |
| *Fusarium fujikuroi* IMI 58289 | 43,832,314 | 12 | 4,234,805 | 65 | 1,182,607 | 3691/14/20 | PRJEB185 |
| *Fusarium verticillioides* 7600 | 41,885,085 | 39 | 1,959,799 | 213 | 392,397 | 3669/24/32 | PRJNA15553 |
| *Fusarium oxysporum* Fo47 | 49,664,628 | 124 | 3,884,136 | 419 | 762,152 | 3688/15/22 | PRJNA67069 |
| *Fusarium oxysporum* f. sp*. lycopersici* 4287 | 61,471,697 | 117 | 1,976,106 | 1,371 | 95,416 | 3598/55/72 | PRJNA18813 |

Table S2. Primers used for gene cloning.

| **Primer** | **Sequence^a^** |
| --- | --- |
| LB019f_538_48_FPOA_13441 | ATGGCGCCTTCAATTATCATACCGTCGTCC |
| LB019r_538_48_FPOA_13441 | TCAAGGAAGCTTCATCATTCGTGTCTCTTTGACC |
| LB020f_538_48_FPOA_13441 | GGCAGCCATATGGCTAGCATGACTGGTGGAATGGCGCCTTCAATTATCATACCGTCGTCC |
| LB020r_538_48_FPOA_13441 | TCTCAGTGGTGGTGGTGGTGGTGCTCGAGTTCAAGGAAGCTTCATCATTCGTGTCTCTTTGACC |

^a^Homology arms of elongated primers for homologous recombination in yeast are underlined.

**Table S3.** List of identified compounds of enzyme reactions.

| **Compound^a^** | ***I*^b^** | ***I* (Lit.)^c^** | **Ident.^d^** |
| --- | --- | --- | --- |
| β-myrcene (**1**) | 992 | 992^2^ | ms, ri |
| β-ocimene (**2**) | 1040 | 1044^3^ | ms, ri |
| monoterpene (**3**) | 1050 |  |  |
| linalool (**4**) | 1100 | 1100^4^ | ms, ri |
| *p*-menth-2-en-1-ol (**5**) | 1124 | 1120^5^ | ms, ri |
| monoterpene (**6**) | 1131 |  |  |
| α-terpineol (**7**) | 1194 | 1189^6^ | ms, ri |
| geraniol (**8**) | 1256 | 1255^7^ | ms, ri, std |
| nerolidol (**11**) | 1568 | 1566^8^ | ms, ri, std |
| koraiol (**9**) | 1632 |  | std |
| farnesol (**10**) | 1727 | 1727^9^ | ms, ri, std |
| diterpene 272 *m*/*z* (**12**) | 2163 |  |  |

^a^Compound numbers refer to compound numbers in Figure S3-S5. ^b^Retention index on a HP5-MS fused silica capillary column. ^c^Retention index on the same or a similar column from tabulated data in the literature. ^d^Identification based on ms: mass spectrum (mass spectral match factor >850), ri: retention index on same or similar column (maximum deviation of 10 points), std: comparison to authentic sample.
